# Forecast Model Analysis for the Morbidity of Tuberculosis in Xinjiang, China

**DOI:** 10.1371/journal.pone.0116832

**Published:** 2015-03-11

**Authors:** Yan-Ling Zheng, Li-Ping Zhang, Xue-Liang Zhang, Kai Wang, Yu-Jian Zheng

**Affiliations:** 1 College of Public Health, Xinjiang Medical University, Urumqi, 830011, People’s Republic of China; 2 College of Medical Engineering and Technology, Xinjiang Medical University, Urumqi, 830011, People’s Republic of China; Fudan University, CHINA

## Abstract

Tuberculosis is a major global public health problem, which also affects economic and social development. China has the second largest burden of tuberculosis in the world. The tuberculosis morbidity in Xinjiang is much higher than the national situation; therefore, there is an urgent need for monitoring and predicting tuberculosis morbidity so as to make the control of tuberculosis more effective. Recently, the Box-Jenkins approach, specifically the autoregressive integrated moving average (ARIMA) model, is typically applied to predict the morbidity of infectious diseases; it can take into account changing trends, periodic changes, and random disturbances in time series. Autoregressive conditional heteroscedasticity (ARCH) models are the prevalent tools used to deal with time series heteroscedasticity. In this study, based on the data of the tuberculosis morbidity from January 2004 to June 2014 in Xinjiang, we establish the single ARIMA (1, 1, 2) (1, 1, 1)_12_ model and the combined ARIMA (1, 1, 2) (1, 1, 1)_12_-ARCH (1) model, which can be used to predict the tuberculosis morbidity successfully in Xinjiang. Comparative analyses show that the combined model is more effective. To the best of our knowledge, this is the first study to establish the ARIMA model and ARIMA-ARCH model for prediction and monitoring the monthly morbidity of tuberculosis in Xinjiang. Based on the results of this study, the ARIMA (1, 1, 2) (1, 1, 1)_12_-ARCH (1) model is suggested to give tuberculosis surveillance by providing estimates on tuberculosis morbidity trends in Xinjiang, China.

## Introduction

Tuberculosis (TB) is a chronic respiratory infectious disease caused by the pathogen Mycobacterium tuberculosis. Infected people can spread TB germs from their mouth when they cough or sneeze. After suffering from TB, if the patients are not given timely, thorough treatment, they can be faced with a serious threat to their health, even making them completely lose the ability to work, but also possibly infecting others. China has a large burden of tuberculosis with huge health and economic losses, the number of TB patients gets the second highest ranking in the world, and around 250 thousand patients die of TB every year [1].

**Fig 1:**
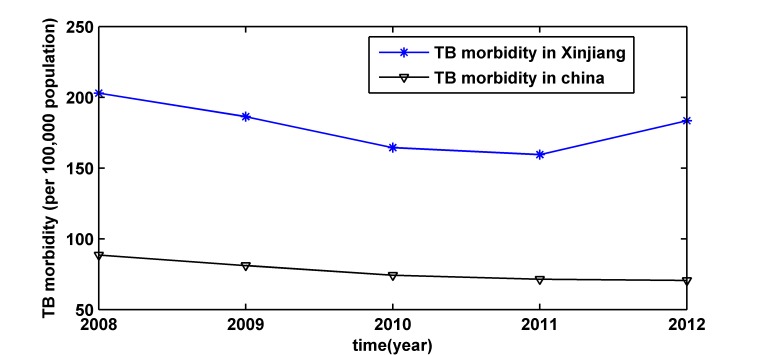
The annual morbidity of tuberculosis from 2008 to 2012 in Xinjiang and in China. Xinjiang is one of the autonomous regions of China; its morbidity of tuberculosis (TB) is much higher than the national situation.

The Xinjiang Uygur Autonomous Region is located in the northwestern border of China; its area is 1.66 million square kilometers, accounting for 1/6 of total area of China, it is the largest autonomous region of China [2]. Its morbidity of TB is much higher than the national situation as shown in Fig. 1. According to the fifth TB epidemiology survey in Xinjiang in 2010, some people over the age of 15 suffered from active pulmonary TB, and the morbidity was 1525 (per 100,000 population), which was 3.32 times higher than the TB morbidity of whole country, the number of active pulmonary TB patients was more than 260,000 [3]. In the last ten years, compared with other infectious disease prevalence, the morbidity of TB has always been ranked in the top two in Xinjiang. This disease is a serious public health and social problem affecting economic and social development, its prevention and control has been signaled as being of great importance: Establishing the accurate morbidity prediction model of TB forecasts future epidemic situation, which can provide scientific basis for formulating the correct control planning.

Some popular methods currently used in prediction for infectious disease morbidity, such as linear regression method [4,5,6], gray model method [7,8,9], artificial neural network method [10,11,12], specifically the autoregressive integrated moving average (ARIMA) method [13,14,15,16,17,18,19], etc. ARIMA method is a reflection of the time dynamic dependency; it can reveal the quantitative relationship between the research object and other objects with the development and change of time. To forecast, ARIMA method is applied more widely than other methods, it can take into account changing trends, periodic changes, and random disturbances in time series, and it is very useful in modeling the temporal dependence structure of a time series.

Some of the morbidity time series, which are difficult to predict accurately with a single model, in this case, some combined models can be used to obtain accurate prediction. For example, Cao et al. [20] used ARIMA-GRNN model to forecast successfully TB incidence in China. Purwanto et al. [21] used ARIMA-NN model to forecast successfully the morbidity of TB in Indonesia and Zambia. YU et al. [22] used a hybrid model with ARIMA and nonlinear autoregressive neural network to forecast successfully incidence cases of hand-foot-mouth disease.

In this study, based on the characteristics of the morbidity of TB in Xinjiang, China, we establish the best single ARIMA model for prediction. In order to improve the accuracy of the single ARIMA model, we make a careful analysis of the residual of the model; we find the residual sequence has heteroscedasticity. Heteroscedasticity is a critical aspect of data non-stationary in time series forecasting, it implies that different observations in time series have different variances. Heteroscedasticity can pose some problems, for example, in the ordinary least squares (OLS) estimate, the presence of heteroscedasticity gives a false sense of precision, and the standard errors and confidence intervals estimated by OLS will be too narrow although the regression coefficients of OLS are still unbiased [23]. Considering this reason, we further establish the autoregressive integrated moving average and autoregressive conditional heteroscedasticity (ARIMA-ARCH) combined model. The results show the accuracy of the combined model is higher than that of single ARIMA model.

## Materials and Methods

### Data Source

The data of the TB cases in Xinjiang from January 2004 to June 2014 was obtained from the website of Bureau of Health, Xinjiang Uyghur Autonomous Region, China, and population data was collected from the Xinjiang statistics Bureau (the calculated TB morbidity in S1 Table). All TB cases were initially diagnosed by clinical symptoms, by bacteriological examination and pathological examination confirmed. Finally, data was collected by case number according to the inspection results. Due to the false negative test results, there might be admission rate bias in the disease report, but this has been reduced as much as possible by repeating test and auxiliary examinations. In China, TB is a nationally notifiable disease and hospital physicians must report every case of TB very seriously to the local health authority within 24 hours. Local health authorities later report monthly TB case totals to higher the national level CDC (Center for Disease Control and Prevention) for surveillance purposes.

### Model Descriptions

**ARIMA Model Description.** The ARIMA model is widely used in the areas of non-stationary time series forecasting, which can be written as:

$$\phi \left(B\right){\left(1-B\right)}^{d}\;X_{t}=\theta \left(B\right)\varepsilon_{t},$$

where *X*_*t*_ represents a non-stationary time series at time *t*, *ε*_*t*_ is a white noise (zero mean and constant variance), *d* is the order of differencing, *B* is a backward shift operator defined by *BX*_*t*_ = *X*_*t*−1_, *φ*(*B*), is the autoregressive operator defined as:

$$\phi \left(B\right)=1-\phi_{1}B-\phi_{2}B^{2}-\cdots -\phi_{p}B^{p},$$

*θ*(*B*) is the moving average operator defined as:

$$\theta \left(B\right)=1-\theta_{1}B-\theta_{2}B^{2}-\cdots -\theta_{q}B^{q}.$$

The periodic repetition of performance norms is very common in time series analyses, this characteristic of many time series is called seasonality, and it is a type of non-stationary. In this case, there are two different components in the ARIMA models: a regular component, which constructs the predictions based on the previous delays in values and disturbances of the variable (with its regular auto regressive (*p*), moving average (*q*), and order of differencing (*d*) components), and a seasonal component, which constructs the predictions based on seasonal delays of values and disturbances of the variable (with its seasonal autoregressive(*P*), moving average (*Q*), and order of differencing (*D*) components). A seasonal ARIMA model with *s* observations per period, denoted by ARIMA (*p*, *d*, *q*) (*P*, *D*, *Q*)_s_ is given by:

$$\Phi \left(B^{s}\right)\phi \left(B\right){\left(1-B\right)}^{d}\;{\left(1-B^{s}\right)}^{D}\;X_{t}=\Theta \left(B^{s}\right)\theta \left(B\right)\varepsilon_{t},$$

$$\Phi \left(B^{s}\right)=1-\phi_{s,1}B^{s}-\phi_{s,2}B^{2s}-\cdots -\phi_{s,P}B^{Ps},$$

$$\Theta \left(B^{s}\right)=1-\theta_{s,1}B^{s}-\theta_{s,2}B^{2s}-\cdots -\theta_{s,Q}B^{Qs}.$$

Generally, the standard statistical methodology to construct an ARIMA model includes four steps:

First step, to transform the non-stationary time series into stationary time series by differencing processes, *d* is the order of non-seasonal (regular) difference, *D* is the order of seasonal difference. Augmented Dickey-Fuller (ADF) test can determine whether the time series after differencing was stationary or not.

Second step, to plot the graphs of the autocorrelation function (ACF) and partial autocorrelation function (PACF) of the transformed series. According to ACF and PACF, we can determine the possible values of *p*, *q*, *P* and *Q*, this process requires both skill and experience. Generally, more than one tentative model is chosen in this step. Then, model identification and parameter estimation.

Third step, to verify the goodness of fit of the possible models by the diagnostic checking of residuals: Residuals must be equivalent to white noises (significant level p>0.05) by using the Box-Jenkins Q test. Generally speaking, if the p value of Q-statistics is not bigger than 0.8, the tentative model is inadequate [24].

Fourth step, to select the best ARIMA model from possible models by the Akaike information criterion (AIC) and Schwarz criterion (SBC) [25].The preferred model is the one with the lowest AIC and SBC values.

**ARIMA-ARCH Model Description.** Autoregressive conditional heteroscedasticity (ARCH) models are the prevalent tools used to deal with time series heteroscedasticity [26]. The error term *ε*_*t*_ of the ARIMA is the random component and commonly assumed to be zero mean and constant variance. However, for some practical time series, the error term *ε*_*t*_ does not satisfy the homoscedastic assumption of constant variance. The time varying variance (i.e., volatility or heteroscedasticity) depends on the observations of the immediate past and is called the conditional variance. In this case, the Histogram-Normality test of the error term *ε*_*t*_ has heavier-tailed distribution [27], as well as, the autoregressive conditional heteroscedasticity Lagrange multiplier (ARCH LM) test of the error term *ε*_*t*_ shows p<0.05. ARCH model is introduced to accommodate the possibility of serial correlation in volatility. Models for volatility forecasting were first developed by Engle (1982) [26], these models known as ARCH models were developed to capture the non-constant variance. Therefore, when the error term *ε*_*t*_ of the ARIMA has ARCH effect, we can consider a combined model, which may have higher accuracy.

The ARIMA-ARCH model is one model, in which the variance of the error term of the ARIMA model follows an ARCH process, the model can be written as [28]:

$$\Phi \left(B^{s}\right)\phi \left(B\right){\left(1-B\right)}^{d}\;{\left(1-B^{s}\right)}^{D}\;X_{t}=\Theta \left(B^{s}\right)\theta \left(B\right)\varepsilon_{t},$$

$$\varepsilon_{t}=\sqrt{v_{t}}z_{t},$$

$$v_{t}=c_{0}+\eta_{1}\varepsilon_{t-1}^{2}+\eta_{2}\varepsilon_{t-2}^{2}+\cdots +\eta_{l}\varepsilon_{t-l}^{2},$$

where the error term *ε*_*t*_ is said to follow an ARCH process of orders *l* [26], [29], *z*_*t*_ is a white noise sequence with mean 0 and variance 1. Assume that *v*_*t*_ is conditioned on the *l* previous errors, *c*_0_ and *η*_i_ are constant coefficients.

### The Indexes of Assessing Forecast Accuracy

Three performance measures were employed in determining prediction efficiency between single ARIMA model and ARIMA-ARCH model, namely root mean square error (RMSE), mean absolute error (MAE) and mean absolute percentage error (MAPE). These measures have been used by many researchers to compare the accuracy of their models with other known models [30, 31, 32, 33, 34].

The first performance measure is root mean square error (RMSE), which is used to compare to predict value with actual value. The RMSE is computed as:

$$\mathrm{RMSE}=\sqrt{\frac{\sum_{t=1}^{n}{\left(X_{t}-{\hat{X}}_{t}\right)}^{2}}{n}}.$$

The second performance measure is mean absolute error (MAE). The MAE is defined as:

$$\mathrm{MAE}=\frac{\sum_{t=1}^{n}\left|X_{t}-{\hat{X}}_{t}\right|}{n}.$$

And then, the third performance measure is mean absolute percentage error (MAPE), a measure of relative overall fitness. This performance measure is defined as:

$$\mathrm{MAPE}=\frac{\sum_{t=1}^{n}\frac{\left|X_{t}-{\hat{X}}_{t}\right|}{X_{t}}\times 100}{n},$$

where *X̂*_*t*_ is the predict value, *X*_*t*_ is the actual value and *n* is the number of observations.

### Data Processing and Analysis

All analyses are performed using Eviews 7.2 and Matlab 2012b.

### Ethical Review

The study protocol and utilization of TB morbidity data were reviewed by Xinjiang Uygur Autonomous Region Center for Disease Control and Prevention and no ethical issues were identified. Therefore, no ethics approval was required by our Investigation Review Board.

## Results

**Fig 2:**
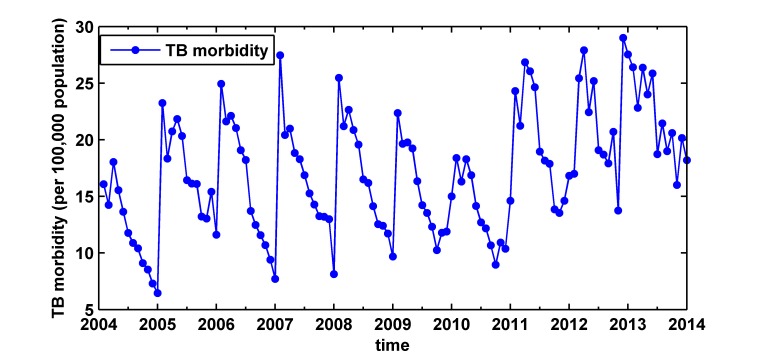
Tuberculosis morbidity from January 2004 to June 2014 in Xinjiang. The Data was obtained from the website of Bureau of Health, Xinjiang Uyghur Autonomous Region, China. The tuberculosis morbidity has roughly seasonal fluctuations and slightly rising trend.

This study is based on the monthly morbidity of TB from January 2004 to June 2014 in Xinjiang, China (as shown in Fig. 2). Fig. 2 shows that the morbidity of TB has roughly seasonal fluctuations and slightly rising trend.

The data set is divided into two subsets for ARIMA model and ARIMA-ARCH model: one for training, and the other one for testing. The data from January 2004 to December 2013 is used to train models, and the data from January 2014 to June 2014 is used to test the performances of the models. ARMA model requires data to be stationary, otherwise, neither of backcast or forecast of the series can be available. Fig. 2 and the ADF test (p>0.05) show the time series is not stationary. In order to obtain a stationary time series, we use three steps to achieve. Firstly, first-order non-seasonal difference (*d* = 1) is computed, after that, ACF and PACF graphs indicate a high seasonal behavior with a circle of 12 (so s = 12), secondly, to remove monthly seasonality, first-order seasonal difference (*D* = 1) with a circle of 12 is computed, finally, to do ADF test, the result (as shown in Table 1) is statistically significant (p<0.001), which confirms that the transformed time series is stationary.

**Table 1:** The ADF test of the transformed tuberculosis morbidity series.

**Covariate**	**t-Statistic**	**p-value**
ADF test statistic	−4.922558	0.0001
1% level statistic	−3.501445	0.01
5% level statistic	−2.892536	0.05
10% level statistic	−2.583371	0.1

After first-order non-seasonal difference and first-order seasonal difference with a circle of 12, we obtain the transformed tuberculosis morbidity series. ADF (Augmented Dickey-Fuller) test statistic is −4.922558, and p = 0.0001 is less than 0.05 significantly, which suggest that the transformed tuberculosis morbidity series is stationary.

**Fig 3:**
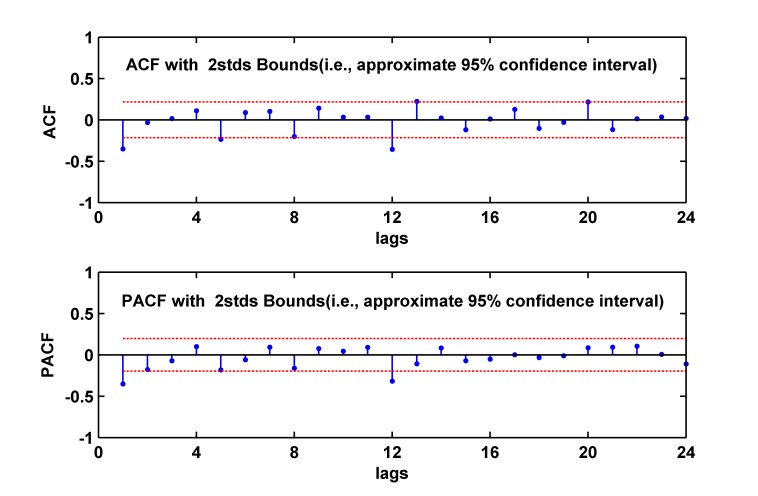
The ACF and PACF graphs of stabilized tuberculosis morbidity series. ACF = autocorrelation function, PACF = partial autocorrelation function. Based on the ACF, we determine the possible values of *q* (*q* = 1, 2 or 3) and *Q*(*Q* = 1) of ARIMA (*p*, *d*, *q*) (*P*, *D*, *Q*) _12_, and based on PACF, we determine the possible values of *p* (*p* = 1, 2 or 3) and *P* (*P* = 1) of ARIMA (*p*, *d*, *q*) (*P*, *D*, *Q*)_12_.

All further statistical procedures are performed on the stationary series. We plot ACF and PACF graphs (as shown in Fig. 3) of the stationary series. By analyzing Fig. 3, we conduct nine models: ARIMA (1, 1, 1) (1, 1, 1)_12_, ARIMA (1, 1, 2) (1, 1, 1)_12_, ARIMA (1, 1, 3) (1, 1, 1)_12_, ARIMA (2, 1, 1) (1, 1, 1)_12_, ARIMA (2, 1, 2) (1, 1, 1)_12_, ARIMA (2, 1, 3) (1, 1, 1)_12_, ARIMA (3, 1, 1) (1, 1, 1)_12_, ARIMA (3, 1, 2) (1, 1, 1)_12_, ARIMA(3, 1, 3)(1, 1, 1)_12_. By diagnostic checking including residual analysis, we establish six models shown in Table 2 with their AIC and SBC, the six models can be used to predict TB morbidity in Xinjiang, China. It is seen from Table 2 that ARIMA (1, 1, 1) (1, 1, 1)_12_ model and ARIMA (1, 1, 2) (1, 1, 1)_12_ model are better than the other four models, since the two models have lower AIC and lower SBC values. Compared with ARIMA (1, 1, 1) (1, 1, 1)_12_ model (the p value of Box-Jenkins Q test is 0.434), the ARIMA (1, 1, 2) (1, 1, 1) _12_ model (the p value of Box-Jenkins Q test is 0.559) has better residual test results, therefore, the ARIMA (1, 1, 2) (1, 1, 1)_12_ model is the better model to fit the data.

**Table 2:** The six ARIMA models with their AIC and SBC values.

**Model**	**AIC**	**SBC**
ARIMA (1, 1, 1) (1, 1, 1)_12_	5.107	5.243
ARIMA (1, 1, 2) (1, 1, 1)_12_	5.09	5.252
ARIMA (2, 1, 1) (1, 1, 1)_12_	5.109	5.273
ARIMA (2, 1, 2) (1, 1, 1)_12_	5.125	5.316
ARIMA (3, 1, 1) (1, 1, 1)_12_	5.136	5.328
ARIMA (3, 1, 2) (1, 1, 1)_12_	5.157	5.377

AIC = Akaike information criterion and SBC = Schwarz criterion.

**Fig 4:**
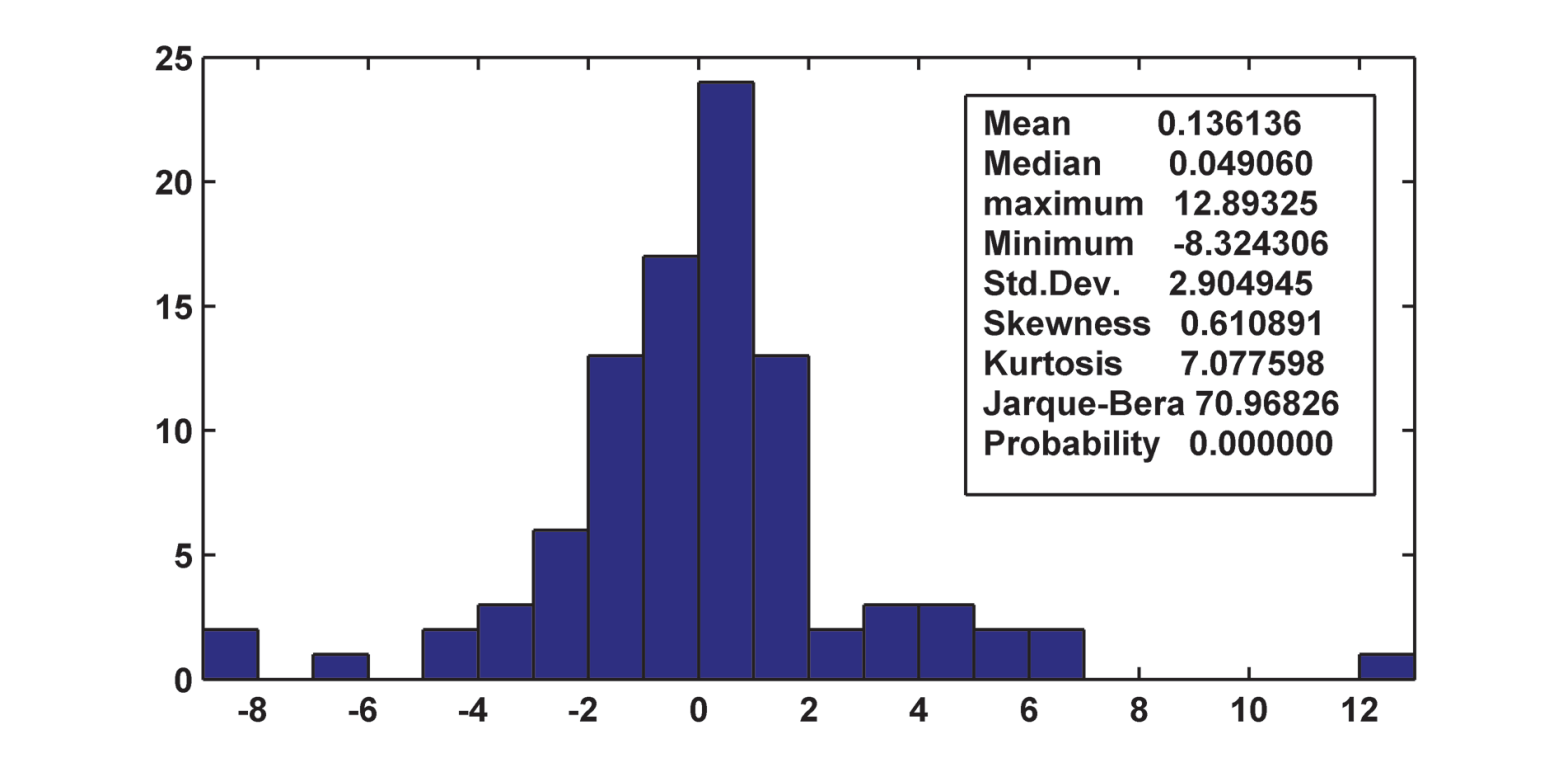
Histogram-Normality test of residual series of the ARIMA (1, 1, 2) (1, 1, 1)_12_ model. Skewness is not 0, Kurtosis is more than 3, Probability is 0.000000, all that suggest the residual series do not obey normal distribution and obey heavier-tailed distribution.

**Fig 5:**
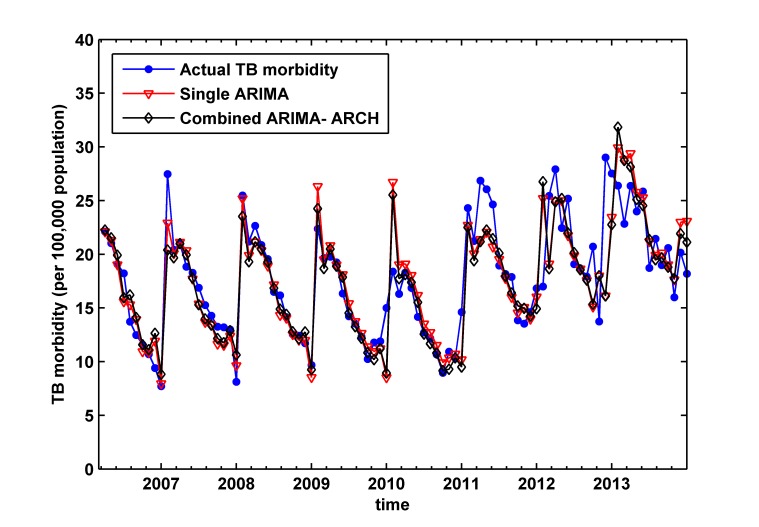
Fitted values of ARIMA (1, 1, 2) (1, 1, 1)_12_ model and ARIMA (1, 1, 2) (1, 1, 1)_12_-ARCH (1) model versus the actual monthly morbidity of tuberculosis before December 2013. We can see fitting performance of the two models by this Figure.

Objective to improve the precision of ARIMA (1, 1, 2) (1, 1, 1)_12_ model, we analyze residual series carefully. Although Box-Jenkins Q test suggest that autocorrelation function of residual series with different lags do not differ from zero (p>0.05), p = 0.559 (corresponding to the *Q*_24_ = 17.457) is not big enough. After that, we do Histogram-Normality test (as shown in Fig. 4), the result shows that heavier-tailed distribution of residual series exists; we do ARCH LM test with the 1st lag, the result shows that a clear ARCH effect of residual series exists (significant level p<0.05), and the ARCH effect do not exist when lag is more than 1. Therefore, we consider establishing ARIMA (1, 1, 2) (1, 1, 1)_12_-ARCH (1) model to improve the precision of prediction. By the diagnostic checking, we find the residual of the combined model is white noise, and there is no ARCH effect longer. The values of AIC and SBC of the combined model (AIC = 4.68 and SBC = 4.92) are less than the corresponding values of single ARIMA (1, 1, 2) (1, 1, 1)_12_ model (AIC = 5.09 and SBC = 5.252), which suggest the proposed combined model is able to achieve significant performance improvement. Fig. 5 shows the actual monthly morbidity of TB and fitted morbidity of ARIMA model and ARIMA-ARCH model.

**Fig 6:**
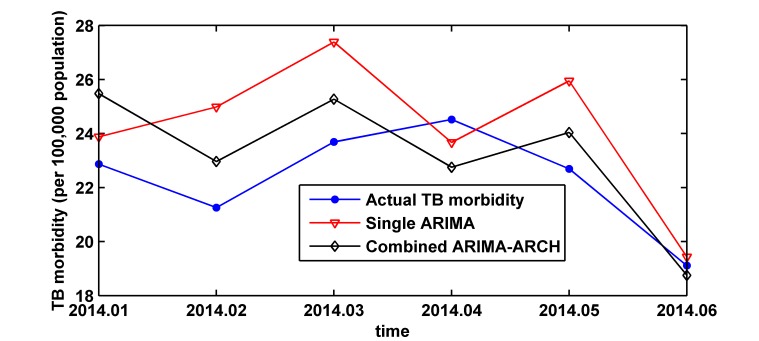
Forecast values of ARIMA (1, 1, 2) (1, 1, 1)_12_ model and ARIMA (1, 1, 2) (1, 1, 1)_12_-ARCH (1) model versus the actual monthly morbidity of tuberculosis from January 2014 to June 2014. We can see predication performance of the two models by this Figure.

Finally, ARIMA model and ARIMA-ARCH model are employed for forecasting TB morbidity from January 2014 to June 2014. The fitting and forecasting results are shown in Fig. 6. The prediction error of the combined model in the testing part is less than the single ARIMA model, as RMSE, MAE and MAPE shown in Table 3, which indicates that the combined model is more effective.

**Table 3:** Forecasting performance comparison by RMSE, MAE and MAPE.

**Model**	**RMSE**	**MAE**	**MAPE**
ARIMA (1, 1, 2) (1, 1, 1)_12_	2.58	2.14	9.51
ARIMA (1, 1, 2) (1, 1, 1)_12_-ARCH (1)	1.7	1.56	6.85

RMSE = root mean square error, MAE = mean absolute error and MAPE = mean absolute percentage error.

## Discussion

TB is a major global public health problem, although substantial progress has been made, it is still an often fatal infectious disease in the world. TB remains a major health issue with a high burden in China. The increase of floating population, TB patients with HIV co-infection as well as the emergence of drug-resistant strains, further lead to the increased difficulty of prevention and control of TB [1]. According to Fig. 1, the TB morbidity in Xinjiang is much higher than the national situation. Fig. 2 shows the morbidity of TB has a slightly rising trend, which indicates there is the bigger challenge for tuberculosis control and prevention. Therefore, it is highly cost effective to detect a TB epidemic in its early stages in order to optimize disease control and intervention in Xinjiang, China. However, up to now, there are no related articles for the prediction of monthly morbidity of TB in Xinjiang. Early warning based on forecasts is very important for improving vector control, community intervention and personal protection. This study aims to develop an appropriate model for predicting TB epidemics in Xinjiang.

Time series analysis of surveillance data on morbidity of various infections is very helpful in developing hypotheses; these hypotheses can explain and anticipate the dynamics of the observed phenomena so as to establish a quality control system. ARIMA model is one of the most widely used time series forecasting techniques because of its structured modeling basis and acceptable forecasting performance [35].

In epidemiology, ARIMA models have been successfully applied to predict the morbidity of infectious disease [13,14,15,16,17,18,19,36,37,38,39]. In this study, the monthly morbidity data of TB from January 2004 to June 2014 was collected in Xinjiang, China. First of all, we use ARIMA method to establish the single ARIMA (1, 1, 2) (1, 1, 1)_12_ model for predicting the monthly morbidity of TB in Xinjiang, after that, we analyze carefully the residual series based on the ARIMA (1, 1, 2) (1, 1, 1)_12_ model, the results indicate that a clear ARCH effect exists. ARCH models are the prevalent tools used to deal with time series heteroscedasticity. In order to remove the heteroscedasticity and improve prediction accuracy, we establish ARIMA (1, 1, 2) (1, 1, 1)_12_-ARCH (1) model. We develop an ARIMA model and an ARIMA-ARCH combined model to predict monthly morbidity of TB in Xinjiang. When we test the performances of the two models based on the data from January 2014 to June 2014, we use three indexes, such as RMSE, MAE and MAPE. The smaller the values of these indexes are, the higher the precision of model is. From Table 3, we can find the combined model, which takes ARCH effect into account, outperform the ARIMA model. We believe that the model combining ARIMA and ARCH effect contains more data characteristics than the single ARIMA (1, 1, 2) (1, 1, 1)_12_ model and is better for forecasting monthly morbidity of TB in Xinjiang.

To the best of our knowledge, this is the first study to establish the ARIMA model and ARIMA-ARCH model for prediction and monitoring the monthly morbidity of TB in Xinjiang. Based on the results of this study, The ARIMA (1, 1, 2) (1, 1, 1)_12_-ARCH (1) model could better contribute to TB surveillance in Xinjiang.

The ARIMA model is generally used for short-term forecasts. Since the morbidity of TB is not stationary, new observations series should be added continually into the sequence over time, which can ensure that the ARIMA-ARCH model provides the best forecast possible. If the actual data falls outside the confidence level of the forecast value, the model should be updated immediately.

## Conclusions

TB is a serious public health issue in Xinjiang, China. Early prediction of TB epidemic is very important for its control and intervention, which can reduce the substantial morbidity and mortality caused by this disease. ARIMA models are an important tool for infectious disease surveillance. ARCH models are the prevalent tools used to deal with time series heteroscedasticity. This study establish ARIMA (1, 1, 2) (1, 1, 1)_12_ model and ARIMA (1, 1, 2) (1, 1, 1)_12_-ARCH (1) model can be employed to forecast the morbidity of TB in Xinjiang, China. Comparative analyses show that the combined model has better performance. Therefore, this study suggests that the department of disease control and prevention uses the ARIMA (1, 1, 2) (1, 1, 1)_12_-ARCH (1) model to optimize TB prevention by providing estimates on TB morbidity trends in Xinjiang, China.

## Supporting Information

S1 Table The data of tuberculosis morbidity in Xinjiang from January 2004 to June 2014. (XLS)
